# Targeted Therapies in Brain Metastases

**DOI:** 10.1007/s11940-013-0276-z

**Published:** 2013-12-19

**Authors:** Nancy U. Lin

**Affiliations:** Department of Medical Oncology, Dana-Farber Cancer Institute, 450 Brookline Avenue, Boston, MA 02215 USA

**Keywords:** Brain metastases, Targeted therapies, Treatment, Breast cancer, Lung cancer, Melanoma, Renal cell carcinoma

## Abstract

Brain metastases are a major clinical problem in patients with advanced breast cancer, lung cancer, melanoma, and renal cell carcinoma. Initial treatment for patients with brain metastases typically includes radiotherapy, either whole brain radiotherapy (WBRT), stereotactic radiosurgery (SRS), or both. Surgical resection is generally reserved for good prognosis patients with limited/controlled extracranial metastases and a single brain lesion. Once patients progress through upfront treatment, the treatment approach is quite variable and there is no clearly defined standard-of-care. Over the past decade, the role of systemic therapies and in particular, targeted therapies has been increasingly explored in patients with brain metastases from solid tumors. For example, lapatinib has been studied as monotherapy, and in combination with capecitabine, in patients with HER2-positive breast cancer, and activity has been observed in both the upfront and refractory settings. In patients with nonsmall cell lung cancer (NSCLC), central nervous system (CNS) activity has been reported with gefinitib and erlotinib. Finally, in melanoma, the B-raf inhibitors vemurafenib and dabrafenib, and the immunomodulator, ipilumimab, have reported CNS activity. Moving forward, the challenge will be to understand how to optimize the activity of targeted agents in the CNS and how to best incorporate them into the current treatment paradigms in order to improve outcomes for this patient population.

## Introduction

Among patients with solid tumors, lung cancer, melanoma, breast cancer, and renal cell carcinoma are most likely to spread to the CNS [1]. For example, over one-quarter of patients with locally advanced or advanced NSCLC will be diagnosed with brain metastases over time [1, 2]. In patients with metastatic HER2-positive breast cancer, the likelihood of eventual CNS involvement is as high as 50 % [3, 4•]. Historically, survival after a diagnosis of CNS metastasis was quite poor [5]. However, recent data indicate favorable trends in survival among some patient subsets [6]. As patients live longer, the need for effective treatments in the upfront and salvage settings has significantly increased as well.

Guidelines for the management of patients with brain metastases have been formulated by several groups, including the European Federation of Neurological Societies (EFNS), National Comprehensive Cancer Network (NCCN), American Society for Radiation Oncology (ASTRO), and the American Association of Neurological Surgeons (AANS)/Congress of Neurological Surgeons [7, 8, 9•, 10]. The choice of therapy depends on a number of factors, including performance status, expected prognosis, number, location, and size of brain metastases, the presence or absence of symptoms and/or mass effect, suitability for surgical resection, and availability (or not) of options to control extracranial disease. A detailed discussion of current management paradigms is outside of the scope of this article. However, the sections below summarize treatment options that are commonly offered today, and provide context for the discussion of targeted systemic therapies to follow.

### Initial management of patients with a single brain metastasis

Patients who present with a single brain lesion should be assessed for their suitability for surgical resection or SRS. Three randomized studies have tested the role of surgical resection followed by WBRT in such patients, compared with WBRT alone. Two of the three studies demonstrated a survival advantage in favor of the surgical arm [11, 12]. A third study was negative, but has been criticized for a relatively high rate of nonadherence to the assigned treatment, as well as enrollment of a patient population with poorer performance status and more active extracranial disease [13]. RTOG 9508 tested the addition of SRS to WBRT in patients with one to three brain metastases [14]. A survival advantage was observed in the subset of patients with a single brain metastasis. Randomized trials directly comparing surgical resection with SRS limited to patients with a single brain metastasis have not been conducted. In the absence of such data, either option may be acceptable in good prognosis patients, though surgical resection is strongly favored when a histological diagnosis is needed, and in the case of large lesions and/or those with significant mass effect. As will be discussed further below, whether or not WBRT should be routinely offered in addition to surgery or SRS is a matter of ongoing debate.

### Initial management of patients with limited brain metastases

Patients who present with limited (ie, two-four) brain metastases may be offered SRS alone, SRS and WBRT, or WBRT alone [9•]. Whether certain histologies should preferentially receive SRS is controversial. Most prospective trials evaluating radiotherapy-based approaches for the treatment of brain metastases have enrolled predominantly patients with NSCLC, with relatively smaller proportions of patients with other tumor types, thus constraining the ability of such trials to answer histology-focused questions [14, 15, 16••]. In a single arm, phase 2 trial of SRS for “radioresistant” histologies (eg, renal cell carcinoma, melanoma, and sarcoma); intracranial failure rates were 25.8 % at 3 months and 48.3 % at 6 months [17].

Several randomized trials have tested the effects of routine WBRT after surgery or SRS on overall survival and cognition, compared with surgery or SRS alone [18]. Aoyama and colleagues randomized patients with one-four brain metastases to SRS alone or SRS plus WBRT [15]. Patients who received SRS alone could later receive WBRT as salvage therapy. Though there was a difference in intracranial control favoring WBRT + SRS, no differences in overall survival were observed, nor were there differences in the likelihood of death due to neurological causes. In terms of neurocognitive outcomes as measured by the Mini-Mental Status Examination (MMSE), the 12-month time point favored WBRT + SRS, likely due to improved intracranial tumor control, though in the small number of patients with extended survival, the 36-month time point showed numerically fewer patients with MMSE decline in the SRS alone arm [19]. The EORTC 22952-26001 trial included patients with one-three brain metastases who could undergo either surgery or radiosurgery, and who were randomized to WBRT or not [16••]. Again, no overall survival improvement was observed with the routine addition of WBRT. Notably, patients who received WBRT experienced worse health related quality of life (HRQOL), particularly during the early follow-up period, relative to patients treated with surgery or SRS alone [20]. Finally, a small study from M.D. Anderson Cancer Center examined the effects of treatment on neurocognitive function [21••]. In this study, patients assigned WBRT + SRS more commonly experienced declines in memory and learning at 4 months, as assessed by the Hopkins Verbal Learning Test, compared with patients who received SRS alone. Curiously, the study also demonstrated a survival advantage for SRS alone. Given that this result was not seen in the much larger randomized trials referenced above, the weight of the evidence does not favor a survival advantage for either strategy.

Given the reproducible reduction in intracranial failure rates, yet the lack of difference in overall survival, individualized discussions with patients are required in making treatment recommendations. In addition, nomograms to predict the risk and timing of subsequent intracranial events would be highly valuable.

### Initial management of patients with multiple brain metastases

For patients who present with multiple brain metastases, WBRT remains the mainstay of therapy [9•, 18]. Best supportive care alone is also an option, particularly in patients with poor performance status. The ongoing QUARTZ trial is a randomized, noninferiority trial investigating whether WBRT adds to best supportive care alone in terms of quality-adjusted life years in patients with inoperable brain metastases from NSCLC. Interim analysis after 151 of 534 planned patients were enrolled demonstrated no decrement in overall survival or quality of life with the omission of WBRT in such patients [22]. At the same time, among patients with breast cancer brain metastases, the number of brain metastases did not appear to be a significant prognostic factor in the analyses leading to the diagnosis-specific graded prognostic assessment (DS-GPA) [23•]. Indeed, in patients with good performance status, and HER2-positive breast cancer, median survival after a brain metastasis diagnosis now approaches 2 years in some series [23•]. These contrasting data highlight some of the disease- and patient-specific considerations in managing patients with brain metastases from solid tumors.

### Management of patients with recurrent/progressive brain metastases

The vast majority of data from high quality, prospective, randomized clinical trials focus on the initial management of patients with brain metastases. There are little to no randomized data to support the choice of one treatment strategy over another in the case of recurrent/progressive CNS disease. The 2013 NCCN guidelines suggest that for patients with one-three metastatic lesions, surgery, SRS, WBRT, or chemotherapy could all be options [8]. For patients with multiple brain metastases who have stable systemic disease or “reasonable” systemic treatment options at the time of CNS progression, the NCCN guidelines state that surgery, re-irradiation, or chemotherapy could all be considered, whereas best supportive care or re-irradiation be considered in patients with systemic disease progression and limited systemic treatment options [8].

## Role of systemic therapy

The role of systemic therapy, either chemotherapy or targeted therapy, in the management of patients with brain metastases is not well-defined. To date, no systemic therapies have gained regulatory approval in the United States for the treatment of brain metastases from solid tumors. However, data supporting the efficacy of systemic therapies is available from prospective clinical trials as well as small experiences in the context of case series or case reports, and significant opportunities exist for drug development in this space [24]. The following section will highlight some of the targeted agents, which have been studied in settings of solid tumor brain metastases.

## Treatment

- The role of systemic therapy in the treatment of patients with solid tumor brain metastases is not well-defined. In particular, the use of systemic therapy in lieu of standard surgical/radiotherapy approaches in newly diagnosed patients is controversial. There are no class I data comparing systemic therapy with localized (ie, surgery/radiotherapy) approaches in this patient population.
- Consideration of systemic therapy should generally occur as part of a multidisciplinary approach, taking into account surgical and radiotherapy options that may also be available.
- Systemic therapy may be an appropriate option in patients who have progressed through standard initial options, such as surgical resection, WBRT, and/or SRS.
- When choosing systemic therapy, the general principles are to prioritize agents with known activity against a specific tumor type, those with evidence of CNS activity, and those with the potential to reach therapeutic levels in brain metastases.
- When available, patients should be considered for clinical trials.
- A comprehensive review of all targeted agents with postulated CNS activity is outside of the scope of this article. However, details of selected agents, prioritizing those with data from prospective clinical trials, are provided below.
- A number of prospective clinical trials of targeted agents are ongoing. Table 1 provides a summary of representative studies.

**Table 1:** Ongoing trials of targeted therapy in patients with solid tumor brain metastases

Agent	Phase of trial	Target	Patient population	ClinicalTrials.gov identifier
Everolimus + trastuzumab + vinorelbine	II	mTOR	HER2+ breast cancer	NCT01305941
BKM120 + trastuzumab	I	PI3K	HER2+ breast cancer	NCT01132664
Lapatinib + WBRT	II	HER2	HER2+ breast cancer	NCT01622868
Neratinib	II	HER2	HER2+ breast cancer	NCT01494662
Afatinib	II	HER2	HER2+ breast cancer	NCT01441596
ARRY-380 + trastuzumab	I	HER2	HER2+ breast cancer	NCT01921335
WBRT +/- erlotinib	II	EGFR	NSCLC	NCT01518621
WBRT + bevacizumab	I	VEGF	Solid tumors	NCT01332929
Bevacizumab	II	VEGF	Solid tumors	NCT01898130
Sunitinib + SRS	I	VEGFR	Solid tumors	NCT00981890
Sorafenib + SRS	I	VEGFR	Solid tumors	NCT01276210
Dabrafenib + SRS	II	BRAF	Melanoma	NCT01721603
Vemurafenib	II	BRAF	Melanoma	NCT01781026
Ipilumumab + WBRT or SRS	I	CLTA-4	Melanoma	NCT01703507
Veliparib + WBRT	II	PARP	NSCLC	NCT01657799

### *Lapatinib*

Lapatinib is an orally bioavailable, dual inhibitor of EGFR and HER2, which is indicated for use in combination with capecitabine for the treatment of patients with advanced or metastatic breast cancer whose tumors overexpress HER2 and who have received prior therapy including an anthracycline, taxane, and trastuzumab. The approval was based on a randomized phase III trial comparing capecitabine vs capecitabine plus lapatinib, and which showed a significant prolongation in time to progression (4.4 months vs 8.4 months; hazard ratio [HR] 0.49 [95 % confidence interval 0.34–0.71], *P* < 0.001) favoring the combination [25]. Of note, patients with active CNS metastases were excluded from the trial, though patients could have been included if their CNS metastases were clinically stable for at least 3 months prior to study entry.

Although lapatinib does not cross the intact blood-brain barrier to a significant degree, it can reach therapeutic levels in brain tumors and brain metastases [26–28]. Data for the use of lapatinib in patients with active brain metastases comes from several prospective clinical trials [29, 30•, 31•, 32]. However, there are not randomized data comparing lapatinib against radiotherapy. EGF105084 was a phase 2, single-arm study evaluating lapatinib monotherapy in 242 patients with HER2-positive breast cancer and progressive brain metastases after WBRT and/or SRS [30•]. The CNS response rate was 6 %. Patients who progressed were allowed to enter an extension arm to receive the combination of lapatinib and capecitabine. Among the 50 evaluable patients who did so, 20 % achieved a CNS objective response. The LANDSCAPE study was a single-arm, phase 2 study, which evaluated the combination of lapatinib and capecitabine in lieu of radiotherapy, in HER2-positive patients with newly diagnosed brain metastases [31•]. The CNS response rate was 65.9 %, and responses were durable. Median TTP in the intent-to-treat population was 5.5 months and 1-year survival exceeded 70 %. Based on the strength of these data, a randomized trial comparing lapatinib and capecitabine vs WBRT is in its planning stages. In addition, trials of other HER2-directed tyrosine kinase inhibitors, including neratinib, afatinib, and ARRY-380 for the treatment of breast cancer brain metastases are currently in progress.

**Standard dosage**: Initiate lapatinib 1,250 mg orally once daily for 21 days in combination with capecitaine 2,000 mg/m2/day (administered orally in two doses approximately 12 hours apart) on days 1–14 of a 21–day cycle. Lapatinib should be taken at least 1 hour before or 1 hour after meals. Capecitabine should be taken with food or within 30 minutes after food.
**Contraindication**: Inability to tolerate or absorb oral medications. Known severe hypersensitivity to either drug or its components. Impaired left ventricular ejection fraction.
**Complications**: Diarrhea, palmar-plantar erythrodysesthesia, acneiform rash, fatigue, hepatic impairment, nausea/vomiting. There is also a small risk of pneumonitis and cardiac impairment [25].
**Special points**: Concomitant use of strong CYP3A4 inhibitors (eg, clarithromycin, ketoconazole) should be avoided. Grapefruit may also increase lapatinib absorption and should be avoided. Patients with baseline severe hepatic dysfunction (Child-Pugh Class C) should begin at a reduced dose of lapatinib.
**Cost/cost-effectiveness**: Expensive.

### *Erlotinib*

Erlotinib is an orally bioavailable EGFR inhibitor, which is indicated for first-line treatment of patients with metastatic NSCLC whose tumors have EGFR exon 19 deletions or exon 21 (L858R) substitution mutations [33•]. The drug is also approved as maintenance therapy in patients whose disease has not progressed after four cycles of platinum-containing first-line therapy.

Evaluation of the efficacy of EGFR inhibitors, including erlotinib and gefitinib, in patients with brain metastases from NSCLC comes from case reports, case series, and small prospective clinical trials [34]. A prospective study of gefitinib in 41 NSCLC patients unselected for mutations status reported an objective response rate of 10 % in the brain [35]. Studies of EGFR inhibitors in patients with known EGFR mutations have demonstrated much higher response rates [36•, 37]. For example, in an open-label phase II study of 28 patients with newly diagnosed NSCLC, measurable brain metastases and a known EGFR mutation, treatment with erlotinib or gefitinib at standard doses resulted in a partial response rate of 83 % and disease control rate of 93 % [36•]. Median progression-free survival (PFS) was 6.6 months and median overall survival was 15.9 months. Of interest, in a nonrandomized retrospective experience, the 1- and 2-year actuarial risk of CNS progression in patients with stage IIIB/IV NSCLC treated with first-line gefitinib or erolotinib was 7 % and 19 %, which is lower than the rate of 40 % expected from historical data [38].

Erlotinib has also been studied in combination with WBRT. In a single-arm phase II trial, forty patients unselected for mutation status received erlotinib 150 mg daily for 1 week, then concurrently with WBRT followed by maintenance [39]. The overall response rate was 86 %. As expected, median survival time was longer among patients with a known EGFR mutation. The Radiation Therapy Oncology Group (RTOG) attempted a phase 3 trial to test the role of temozolomide or erlotinib in addition to WBRT + SRS in patients with NSCLC and up to three brain metastases [40]. The studied closed early after 126 patients were enrolled due to slow accrual. At the time of analysis, there were not significant differences in survival seen between arms, and if anything, the numerical trends favored the WBRT + SRS only arm, possibly due to increased toxicity in the combination arm. Thus, at this time, concurrent erlotinib with radiotherapy is not recommended outside of a clinical trial. Indeed, the data in EGFR-mutant patients treated with erlotinib alone, as detailed above, raise the question of whether it should be the preferred front-line approach, in lieu of radiotherapy, with radiotherapy reserved for salvage, particularly in patients presenting with asymptomatic brain metastases.

Pulsatile dosing of EGFR tyrosine kinase inhibitors has also been explored as a way to improve the CNS penetration of the drug [41, 42]. In nine patients who had developed parenchymal or leptomeningeal metastases on standard dose erlotinib or other EGFR tyrosine kinase inhibitors, six (67 %, including two patients with leptomeningeal disease) responded to erlotinib when given at a dose of 1,500 mg once weekly.

**Standard dosage**: 150 mg orally once daily, taken at least 1 hour before or 2 hours after a meal.
**Contraindication**: Inability to tolerate or absorb oral medications.
**Complications**: Diarrhea, acneiform rash, anorexia, fatigue, dyspnea, cough, nausea, vomiting. Interstitial lung disease in about 1 % of patients.
**Special points**: Concomitant use of strong CYP3A4 inhibitors (eg, clarithromycin, ketoconazole) should be avoided. Grapefruit may also increase erlotinib absorption and should be avoided.
**Cost/cost-effectiveness**: Expensive.

### *Vemurafenib*

Vemurafenib is an oral BRAF inhibitor indicated in patients with unresectable or metastatic melanoma whose tumors contain the BRAF V600E mutation. Approval was based on a randomized trial comparing vemurafenib with dacarbazine, which showed a statistically significant improvement in progression-free and overall survival [43••].Of note, patients with CNS metastases were excluded from the pivotal trial, unless the CNS disease had been definitively treated more than 3 months previously with no evidence of progression and no requirement for ongoing glucocorticoid treatment.

Preliminary results have been reported from a single-arm pilot study evaluating vemurafenib in 24 patients with V600E-mutated melanoma and nonresectable brain metastases pretreated with radiotherapy and/or chemotherapy [44]. Partial responses in both body and brain have been noted. At the same time, there have also been case reports of isolated CNS progression in the setting of extracranial disease response, raising questions about CNS penetration and relative CNS response compared with extracranial response [45]. Vemurafenib has not been directly compared with radiotherapy-based approaches for the treatment of melanoma brain metastases.

**Standard dosage**: 960 mg orally twice daily, administered approximately 12 hours apart, with or without a meal.
**Contraindication**: None.
**Complications**: The most common adverse reactions are arthralgia, rash, alopecia, fatigue, photosensitivity reaction, nausea, pruritus, and skin papilloma.
**Special points**: Cutaneous squamous cell carcinomas occur in about one-quarter of patients. Baseline and follow-up skin examinations should be performed for patients receiving vemurafenib. QT prolongation has also been reported and drug should be held if the QTc exceeds 500 ms. Uveitis and iritis can occur. Patients should be monitored for visual symptoms.
**Cost/cost-effectiveness**: Expensive.

### *Dabrafenib*

Dabrafenib is an oral BRAF inhibitor indicated in patients with unresectable or metastatic melanoma whose tumors contain the BRAF V600E mutation. Approval was based on a randomized trial comparing dabrafenib with dacarbazine, which showed a statistically significant improvement in objective response rate and progression-free survival [46••].

Dabrafenib has been studied in a phase 1 dose-escalation trial, which included ten patients with untreated melanoma brain metastases [47]. In this study, nine of ten patients experienced a reduction in the size of their brain lesions. A subsequent multicenter phase 2 trial enrolled 172 patients with V600E or V600K BRAF-mutant melanoma and either untreated (Cohort A) or previously treated (Cohort B) asymptomatic brain metastases [48]. Intracranial responses by modified RECIST criteria were noted in both cohorts (for V600E, 39.2 % in Cohort A and 30.8 % in Cohort B; for V600K, 6.7 % in Cohort A and 22.2 % in Cohort B). Intracranial hemorrhage was reported in 6 % of patients enrolled on the study. As with vemurafenib, trials directly comparing dabrafenib against radiotherapy have not been conducted.

**Standard dosage**: 150 mg orally twice daily, at least 1 hour before or 2 hours after a meal.
**Contraindication**: None.
**Complications**: The most common adverse reactions are hyperkeratosis, headache, pyrexia, arthralgia, papilloma, alopecia, and palmar-plantar erythrodysesthesia.
**Special points**: Cutaneous squamous cell carcinomas occur in about one-quarter of patients. Baseline and follow-up skin examinations should be performed for patients receiving dabrafenib. Uveitis and iritis can occur. Patients should be monitored for visual symptoms. Febrile drug reactions can occur and may necessitate drug hold or discontinuation.
**Cost/cost-effectiveness**: Expensive.

### *Ipilumumab*

Ipilumumab is a monoclonal antibody directed against cytotoxic T-lymphocyte antigen-4 (CTLA-4). It is indicated for the treatment of unresectable or metastatic melanoma based on results of a randomized trial demonstrating an overall survival advantage compared with a tumor vaccine or vaccine placebo in patients who had previously received at least one prior systemic treatment [49].

Ipilumumab has also been studied in an open-label phase 2 trial in patients with melanoma brain metastases [50]. Among 51 patients with asymptomatic brain metastases on study entry, nine patients (18 %) exhibited disease control in both brain and body. Among 21 patients who were symptomatic and on corticosteroids, one patient (5 %) exhibited disease control in all sites. Whether ipilumumab administration after SRS provides clinical benefit is unclear. One small retrospective study including 25 patients treated with ipilumumab found no difference in the rate of freedom from new brain metastases or overall survival compared with patients who received SRS alone [51]. However, another retrospective study did appear to support a possible improvement in survival [52]. Whether newer immunomodulatory approaches, such as PD-1 and PD-L1 inhibitors will have CNS activity remains to be seen.

**Standard dosage**: Three mg/kg as an intravenous infusion every 3 weeks for a total of four doses.
**Contraindication**: None.
**Complications**: The most common adverse events are immune-mediated reactions, such as diarrhea, pruritus, rash, and colitis.
**Special points**: Ipilumumab can result in severe and fatal immune-mediated adverse reactions, which can involve any organ system. These can include enterocolitis, hepatitis, dermatitis, neuropathy, and endocrinopathy. Patients should be assessed at baseline and before each dose. Ipilumumab should be permanently discontinued for severe immune-mediated reactions.
**Cost/cost-effectiveness**: Expensive.

### *Bevacizumab*

Bevacizumab is a humanized monoclonal antibody directed against vascular endothelial growth factor (VEGF)-A. It is currently approved by the United States Food and Drug Administration (FDA) for the treatment of several malignancies, including metastatic colorectal cancer, metastatic renal cell carcinoma, and NSCLC. The breast cancer approval was revoked in 2011.

In the majority of the initial trials of bevacizumab, patients with brain metastases, whether stable or active, were excluded out of a concern for intracranial hemorrhage. More recent data supports the safety of bevacizumab in patients with both treated and untreated brain metastases. A retrospective exploratory analysis from 13 randomized controlled trials included 131 patients with treated CNS metastases who went on to receive bevacizumab. Only one patient (0.8 %) developed grade 2 cerebral hemorrhage [53]. The PASSPORT trial enrolled 115 patients with treated brain metastases and reported no episodes of grade 2 or higher CNS hemorrhage [54].

Data supporting the potential for clinical activity of bevacizumab in patients with active brain metastases comes from case series and small prospective trials. In breast cancer, a small series (*n* = 4) of patients were treated with the combination of bevacizumab and paclitaxel. All patients responded (1 CR, 3 PR) with duration of response 6 to 11 months [55]. Combinations of bevacizumab and platinum agents have been studied in two prospective trials, both of which are available in abstract form [56, 57]. The larger of the two studies reported a CNS response rate of 63 % (95 % CI 46 %–78 %) among 38 patients with either HER2-negative or HER2-positive breast cancer [57]. A randomized trial (with an overall survival endpoint) to test the role of bevacizumab in breast cancer patients is being considered. Anecdotal evidence of CNS activity has also been reported in NSCLC [58]. As in breast cancer, randomized data are not available to fully assess its clinical impact.

**Standard dosage**: Dose and schedule vary by indication. Please refer to package insert for details.
**Contraindication**: Do not initiate bevacizumab for 28 days following major surgery and until surgical wound is fully healed. Do not administer bevacizumab to patients with serious hemorrhage or recent hemoptysis.
**Complications**: The most common adverse reactions include epistaxis, headache, hypertension, rhinitis, and proteinuria. The incidence of gastrointestinal perforation is 0.3 %–2.5 %. Bevacizumab increases the risk for both bleeding as well as thromboembolic events. Rarely, reversible posterior leukoencephalopathy (RPLS) may occur.
**Special points**: None.
**Cost/cost-effectiveness**: Expensive.

### *Sunitinib*

Sunitinib is an oral inhibitor of VEGFR, PDGFR, and c-kit indicated for the treatment of advanced renal cell carcinoma (RCC). Approval was based on an improvement in PFS compared with interferon-α when given in the first-line setting [59]. Notably, patients with brain metastases were excluded from the pivotal trial.

Subsequently, 321 patients with brain metastases were treated as part of an expanded access program [60]. In this experience, objective responses were observed in 26/213 (12 %) of evaluable patients. Median PFS was 5.6 months. The toxicity profile was comparable with that of the general metastatic RCC population. Only one patient experienced a grade 1/2 cerebral hemorrhage. No grade 3 or 4 CNS hemorrhage events were observed. Other groups have since corroborated these findings, albeit with small numbers of treated patients [61, 62].

**Standard dosage**: For RCC, 50 mg orally once daily, with or without food, in a 6-week cycle (4 weeks on-treatment / 2 weeks off)
**Contraindication**: None.
**Complications**: The most common adverse reactions are fatigue, asthenia, fever, diarrhea, nausea, mucositis/stomatitis, vomiting, dyspepsia, abdominal pain, constipation, hypertension, peripheral edema, rash, palmar-plantar erythrodysesthesia, skin discoloration, dry skin, hair color changes, altered taste, headache, back pain, arthralgia, extremity pain, cough, dyspnea, anorexia, and bleeding.
**Special points**: Hepatotoxicity has been observed in clinical trials and postmarketing experience. This hepatotoxicity may be severe, and deaths have been reported. Liver function tests should be checked at baseline and monitored during each cycle of treatment. Cardiac toxicity has been observed. Prolonged QT intervals have been observed. Thyroid dysfunction may occur. Consider dose reduction when administered with strong CYP3A4 inhibitors.
**Cost/cost-effectiveness**: Expensive.

### *Sorafenib*

Sorafenib is an oral VEGF receptor and Raf kinase inhibitor indicated for the treatment of patients with advanced RCC. Approval was based on a randomized phase III study demonstrating a PFS benefit compared with placebo in patients who had received at least one prior systemic treatment [63]. Patients with brain metastases were not eligible for trial participation.

In the expanded access program, patients with brain metastases were allowed. In the brain metastasis subset (*n* = 70), two patients (4 %) achieved a partial response, and 34 patients (68 %) experienced stable disease for at least 8 weeks [64]. Among the program as a whole (*n* = 2,504), CNS hemorrhage occurred in less than 1 % of patients; no cases of CNS hemorrhage were noted in the brain metastasis subset. The effect of sorafenib on the incidence of brain metastases has also been studied. In a post-hoc analysis of patients enrolled on the Treatment Approaches in Renal Cancer Global Evaluation Trial (TARGET), the incidence of brain metastases was lower in patients who received sorafenib compared with patients who received placebo (3 % vs 12 %, *P* < 0.05), raising the question of whether targeted agents might be used as chemoprevention [65]. Studies directly comparing radiotherapy vs sorafenib have not been reported.

**Standard dosage**: 400 mg orally twice daily taken either 1 hour before or 2 hours after meals.
**Contraindication**: Known severe hypersensitivity to sorafenib or any other component of sorafenib.
**Complications**: Adverse events include fatigue, weight loss, rash, palmar-plantar erythrodysesthesia, diarrhea, anorexia, and hypertension. There is an increase in the risk of cardiac ischemic events (2.9 % vs 0.4 % in the placebo group). Asymptomatic hypophosphatemia and elevated serum lipase are common.
**Special points**: Hypertension is common and usually occurs early in the course of treatment. Blood pressure should be monitored weekly during the first cycle and periodically thereafter. Sorafenib should be held in patients undergoing major surgical procedures.
**Cost/cost-effectiveness**: Expensive.

## References

[1] Barnholtz-Sloan JS, Sloan AE, Davis FG, Vigneau FD, Lai P, Sawaya RE (2004). Incidence proportions of brain metastases in patients diagnosed (1973 to 2001) in the Metropolitan Detroit Cancer Surveillance System. J Clin Oncol.

[2] Gaspar LE, Chansky K, Albain KS, Vallieres E, Rusch V, Crowley JJ (2005). Time from treatment to subsequent diagnosis of brain metastases in stage III nonsmall-cell lung cancer: a retrospective review by the Southwest Oncology Group. J Clin Oncol.

[3] Olson EM, Najita JS, Burstein HJ, Sohl J, Arnaout A, Winer EP, et al. Clinical outcomes and treatment practice patterns of patients with HER2-positive metastatic breast cancer in the post-trastuzumab era. The Breast. 2013;22(4):525–31. 10.1016/j.breast.2012.12.006 PMC3713786 23352568

[4.•] Pestalozzi BC, Holmes E, de Azambuja E, Metzger-Filho O, Hogge L, Scullion M (2013). CNS relapses in patients with HER2-positive early breast cancer who have and have not received adjuvant trastuzumab: a retrospective substudy of the HERA trial (BIG 1-01). Lancet Oncol.

[5] Borgelt B, Gelber R, Kramer S, Brady LW, Chang CH, Davis LW (1980). The palliation of brain metastases: final results of the first two studies by the Radiation Therapy Oncology Group. Int J Radiat Oncol Biol Phys.

[6] Sperduto PW, Kased N, Roberge D, Xu Z, Shanley R, Luo X, et al. Effect of tumor subtype on survival and the graded prognostic assessment for patients with breast cancer and brain metastases. Int J Radiat Oncol Biol Phys. 2012;82(5):2111–7. 10.1016/j.ijrobp.2011.02.027 PMC3172400 21497451

[7] Soffietti R, Cornu P, Delattre JY, Grant R, Graus F, Grisold W (2006). EFNS Guidelines on diagnosis and treatment of brain metastases: report of an EFNS Task Force. Eur J Neurol.

[8] National Comprehensive Cancer Network (NCCN) clinical practice guidelines in oncology. Central Nervous System Cancers. 2013. Available at: www.nccn.org/professionals/physician_gls/pdf/cns. Accessed 1 Aug 2013.

[9.•] Tsao MN, Rades D, Wirth A, Lo SS, Danielson BL, Gaspar LE (2012). Radiotherapeutic and surgical management for newly diagnosed brain metastasis(es): an American Society for Radiation Oncology evidence-based guideline. Pract Radiat Oncol..

[10] Linskey ME, Kalkanis SN (2010). Evidence-linked, clinical practice guidelines-getting serious; getting professional. J Neuro Oncol.

[11] Patchell RA, Tibbs PA, Walsh JW, Dempsey RJ, Maruyama Y, Kryscio RJ (1990). A randomized trial of surgery in the treatment of single metastases to the brain. N Engl J Med.

[12] Noordijk EM, Vecht CJ, Haaxma-Reiche H, Padberg GW, Voormolen JH, Hoekstra FH (1994). The choice of treatment of single brain metastasis should be based on extracranial tumor activity and age. Int J Radiat Oncol Biol Phys.

[13] Mintz AH, Kestle J, Rathbone MP, Gaspar L, Hugenholtz H, Fisher B (1996). A randomized trial to assess the efficacy of surgery in addition to radiotherapy in patients with a single cerebral metastasis. Cancer.

[14] Andrews DW, Scott CB, Sperduto PW, Flanders AE, Gaspar LE, Schell MC (2004). Whole brain radiation therapy with or without stereotactic radiosurgery boost for patients with one to three brain metastases: phase III results of the RTOG 9508 randomised trial. Lancet.

[15] Aoyama H, Shirato H, Tago M, Nakagawa K, Toyoda T, Hatano K (2006). Stereotactic radiosurgery plus whole-brain radiation therapy vs stereotactic radiosurgery alone for treatment of brain metastases: a randomized controlled trial. JAMA.

[16.••] Kocher M, Soffietti R, Abacioglu U, Villa S, Fauchon F, Baumert BG (2011). Adjuvant whole-brain radiotherapy vs observation after radiosurgery or surgical resection of one to three cerebral metastases: results of the EORTC 22952-26001 study. J Clin Oncol.

[17] Manon R, O'Neill A, Knisely J, Werner-Wasik M, Lazarus HM, Wagner H (2005). Phase II trial of radiosurgery for one to three newly diagnosed brain metastases from renal cell carcinoma, melanoma, and sarcoma: an Eastern Cooperative Oncology Group study (E 6397). J Clin Oncol.

[18] Tsao MN, Lloyd N, Wong RK, Chow E, Rakovitch E, Laperriere N, et al. Whole brain radiotherapy for the treatment of newly diagnosed multiple brain metastases. Cochrane Database of Systematic Reviews (Online). 2012;4:CD003869. 10.1002/14651858.CD003869.pub3 PMC6457607 22513917

[19] Aoyama H, Tago M, Kato N, Toyoda T, Kenjyo M, Hirota S (2007). Neurocognitive function of patients with brain metastasis who received either whole brain radiotherapy plus stereotactic radiosurgery or radiosurgery alone. Int J Radiat Oncol Biol Phys.

[20] Soffietti R, Kocher M, Abacioglu UM, Villa S, Fauchon F, Baumert BG (2013). A European organisation for research and treatment of cancer phase iii trial of adjuvant whole-brain radiotherapy versus observation in patients with one to three brain metastases from solid tumors after surgical resection or radiosurgery: quality-of-life results. J Clin Oncol.

[21.••] Chang EL, Wefel JS, Hess KR, Allen PK, Lang FF, Kornguth DG, et al. Neurocognition in patients with brain metastases treated with radiosurgery or radiosurgery plus whole-brain irradiation: a randomized controlled trial. Lancet Oncol. 2009;10(11):1037–44. This study assessed the impact of WBRT in patients treated with radiosurgery using a battery of validated neurocognitive function tests and demonstrated decrements in neurocognition at the pre-specified 4 month time point. Though small, the study has had a significant impact on the debate regarding the value of initial WBRT in patients with a limited number of brain metastases. 10.1016/S1470-2045(09)70263-3 19801201

[22] Langley RE, Stephens RJ, Nankivell M, Pugh C, Moore B, Navani N (2013). Interim data from the Medical Research Council QUARTZ Trial: does whole brain radiotherapy affect the survival and quality of life of patients with brain metastases from nonsmall cell lung cancer?. Clin Oncol.

[23.•] Sperduto PW, Kased N, Roberge D, Xu Z, Shanley R, Luo X, et al. Summary Report on the Graded Prognostic Assessment: an accurate and facile diagnosis-specific tool to estimate survival for patients with brain metastases. J Clin Oncol. 2012;30(4):419–25. This publication summarizes the diagnosis-specific prognostic tools which can be used to estimate survival after a brain metastasis diagnosis in the major primary tumor types. 10.1200/JCO.2011.38.0527 PMC3269967 22203767

[24] Preusser M, Berghoff AS, Schadendorf D, Lin NU, Stupp R (2012). Brain metastasis: opportunity for drug development?. Curr Op Neurol.

[25] Geyer CE, Forster J, Lindquist D, Chan S, Romieu CG, Pienkowski T (2006). Lapatinib plus capecitabine for HER2-positive advanced breast cancer. N Engl J Med.

[26] Kuhn J, Robins HI, Mehta M, Fine H, Cloughesy T, Wen P, et al. Tumor sequestration of lapatinib (NABTC 04-01). Neuro Oncol. 2008;10(5): [Abstract ET-05].

[27] Morikawa A, Peereboom DM, Smith QR, Thorsheim H, Lockman PR, Simmons AJ, et al. Clinical evidence for drug penetration of capecitabine and lapatinib uptake in resected brain metastases from women with metastatic breast cancer. J Clin Oncol. 2013: [Abstract 514].

[28] Taskar KS, Rudraraju V, Mittapalli RK, Samala R, Thorsheim HR, Lockman J (2012). Lapatinib distribution in HER2 overexpressing experimental brain metastases of breast cancer. Pharm Res.

[29] Lin NU, Carey LA, Liu MC, Younger J, Come SE, Ewend M (2008). Phase II trial of lapatinib for brain metastases in patients with human epidermal growth factor receptor 2-positive breast cancer. J Clin Oncol.

[30.•] Lin NU, Dieras V, Paul D, Lossignol D, Christodoulou C, Stemmler HJ (2009). Multicenter phase II study of lapatinib in patients with brain metastases from HER2-positive breast cancer. Clin Cancer Res.

[31.•] Bachelot T, Romieu G, Campone M, Dieras V, Cropet C, Dalenc F (2013). Lapatinib plus capecitabine in patients with previously untreated brain metastases from HER2-positive metastatic breast cancer (LANDSCAPE): a single-group phase 2 study. Lancet Oncol.

[32] Lin NU, Eierman W, Greil R, Campone M, Kaufman B, Steplewski K, et al. Randomized phase II study of lapatinib plus capecitabine or lapatinib plus topotecan for patients with HER2-positive breast cancer brain metastases. J Neuro Oncol. 2011;105(3):613–20. 10.1007/s11060-011-0629-y 21706359

[33.••] Rosell R, Carcereny E, Gervais R, Vergnenegre A, Massuti B, Felip E (2012). Erlotinib vs standard chemotherapy as first-line treatment for European patients with advanced EGFR mutation-positive nonsmall-cell lung cancer (EURTAC): a multi-center, open-label, randomized phase 3 trial. Lancet Oncol.

[34] Jamal-Hanjani M, Spicer J (2012). Epidermal growth factor receptor tyrosine kinase inhibitors in the treatment of epidermal growth factor receptor-mutant nonsmall cell lung cancer metastatic to the brain. Clin Cancer Res.

[35] Ceresoli GL, Cappuzzo F, Gregorc V, Bartolini S, Crino L, Villa E (2004). Gefitinib in patients with brain metastases from nonsmall-cell lung cancer: a prospective trial. Ann Oncol.

[36.•] Park SJ, Kim HT, Lee DH, Kim KP, Kim SW, Suh C (2012). Efficacy of epidermal growth factor receptor tyrosine kinase inhibitors for brain metastasis in nonsmall cell lung cancer patients harboring either exon 19 or 21 mutation. Lung Cancer.

[37] Wu YL, Zhou C, Cheng Y, Lu S, Chen GY, Huang C (2013). Erlotinib as second-line treatment in patients with advanced nonsmall-cell lung cancer and asymptomatic brain metastases: a phase II study (CTONG-0803). Ann Oncol.

[38] Heon S, Yeap BY, Britt GJ, Costa DB, Rabin MS, Jackman DM (2010). Development of central nervous system metastases in patients with advanced nonsmall cell lung cancer and somatic EGFR mutations treated with gefitinib or erlotinib. Clin Cancer Res.

[39] Welsh JW, Komaki R, Amini A, Munsell MF, Unger W, Allen PK (2013). Phase II trial of erlotinib plus concurrent whole-brain radiation therapy for patients with brain metastases from nonsmall-cell lung cancer. J Clin Oncol.

[40] Sperduto PW, Wang M, Robins HI, Schell MC, Werner-Wasik M, Komaki R (2013). A phase 3 trial of whole brain radiation therapy and stereotactic radiosurgery alone vs WBRT and SRS with temozolomide or erlotinib for nonsmall cell lung cancer and 1 to 3 brain metastases: Radiation Therapy Oncology Group 0320. Int J Radiat Oncol Biol Phys.

[41] Jackman DM, Holmes AJ, Lindeman N, Wen PY, Kesari S, Borras AM (2006). Response and resistance in a nonsmall-cell lung cancer patient with an epidermal growth factor receptor mutation and leptomeningeal metastases treated with high-dose gefitinib. J Clin Oncol.

[42] Grommes C, Oxnard GR, Kris MG, Miller VA, Pao W, Holodny AI (2011). "Pulsatile" high-dose weekly erlotinib for CNS metastases from EGFR mutant nonsmall cell lung cancer. Neuro Oncol..

[43.••] Chapman PB, Hauschild A, Robert C, Haanen JB, Ascierto P, Larkin J (2011). Improved survival with vemurafenib in melanoma with BRAF V600E mutation. N Engl J Med.

[44] Dummer R, Rinderknecht J, Goldinger SM, Wagner I, Mitchell L, Veronese ML, et al. An open-label pilot study of vemurafenib in previously treated metastatic melanoma patients with brain metastases. J Clin Oncol. 2011;29(Suppl): [Abstract 8548].

[45] Rochet NM, Dronca RS, Kottschade LA, Chavan RN, Gorman B, Gilbertson JR (2012). Melanoma brain metastases and vemurafenib: need for further investigation. Mayo Clin Proc.

[46.••] Hauschild A, Grob JJ, Demidov LV, Jouary T, Gutzmer R, Millward M (2012). Dabrafenib in BRAF-mutated metastatic melanoma: a multi-center open-label, phase 3 randomized controlled trial. Lancet.

[47] Falchook GS, Long GV, Kurzrock R, Kim KB, Arkenau TH, Brown MP (2012). Dabrafenib in patients with melanoma, untreated brain metastases, and other solid tumours: a phase 1 dose-escalation trial. Lancet.

[48] Long GV, Trefzer U, Davies MA, Kefford RF, Ascierto PA, Chapman PB (2012). Dabrafenib in patients with Val600Glu or Val600Lys BRAF-mutant melanoma metastatic to the brain (BREAK-MB): a multi-center, open-label, phase 2 trial. Lancet Oncol.

[49] Hodi FS, O'Day SJ, McDermott DF, Weber RW, Sosman JA, Haanen JB (2010). Improved survival with ipilimumab in patients with metastatic melanoma. N Engl J Med.

[50] Margolin K, Ernstoff MS, Hamid O, Lawrence D, McDermott D, Puzanov I (2012). Ipilimumab in patients with melanoma and brain metastases: an open-label, phase II trial. Lancet Oncol.

[51] Mathew M, Tam M, Ott PA, Pavlick AC, Rush SC, Donahue BR (2013). Ipilimumab in melanoma with limited brain metastases treated with stereotactic radiosurgery. Melanoma Res.

[52] Knisely JP, Yu JB, Flanigan J, Sznol M, Kluger HM, Chiang VL (2012). Radiosurgery for melanoma brain metastases in the ipilimumab era and the possibility of longer survival. J Neurosurg.

[53] Besse B, Lasserre SF, Compton P, Huang J, Augustus S, Rohr UP (2010). Bevacizumab safety in patients with central nervous system metastases. Clin Cancer Res.

[54] Socinski MA, Langer CJ, Huang JE, Kolb MM, Compton P, Wang L (2009). Safety of bevacizumab in patients with nonsmall-cell lung cancer and brain metastases. J Clin Oncol.

[55] Labidi SI, Bachelot T, Ray-Coquard I, Mosbah K, Treilleux I, Fayette J (2009). Bevacizumab and paclitaxel for breast cancer patients with central nervous system metastases: a case series. Clin Breast Cancer.

[56] Lu YS, Chen WW, Ling CH, Tseng LM, Yeh DC, Wu PF, et al. Bevacizumab, etoposide, and cisplatin (BEEP) in brain metastases of breast cancer progressing from radiotherapy: results of the first stage of a multicenter phase II study. J Clin Oncol. 2012;30: [Abstract 1079].

[57] Lin NU, Gelman R, Younger J, Sohl J, Freedman R, Sorensen AG, et al. Phase II Trial of Carboplatin (C) and Bevacizumab (BEV) in Patients (Pts) with Breast Cancer Brain Metastases (BCBM). J Clin Oncol. 2013;31: [Abstract 513].

[58] De Braganca KC, Janjigian YY, Azzoli CG, Kris MG, Pietanza MC, Nolan CP (2010). Efficacy and safety of bevacizumab in active brain metastases from nonsmall cell lung cancer. J Neuro Oncol.

[59] Motzer RJ, Hutson TE, Tomczak P, Michaelson MD, Bukowski RM, Rixe O (2007). Sunitinib vs interferon alfa in metastatic renal-cell carcinoma. N Engl J Med.

[60] Gore ME, Hariharan S, Porta C, Bracarda S, Hawkins R, Bjarnason GA (2011). Sunitinib in metastatic renal cell carcinoma patients with brain metastases. Cancer.

[61] Choueiri TK, Duh MS, Clement J, Brick AJ, Rogers MJ, Kwabi C (2010). Angiogenesis inhibitor therapies for metastatic renal cell carcinoma: effectiveness, safety and treatment patterns in clinical practice-based on medical chart review. BJU Int.

[62] Lim ZD, Mahajan A, Weinberg J, Tannir NM (2013). Outcome of patients with renal cell carcinoma metastatic to the brain treated with sunitinib without local therapy. Am J Clinl Oncol.

[63] Escudier B, Eisen T, Stadler WM, Szczylik C, Oudard S, Siebels M (2007). Sorafenib in advanced clear-cell renal-cell carcinoma. N Engl J Med.

[64] Stadler WM, Figlin RA, McDermott DF, Dutcher JP, Knox JJ, Miller WH (2010). Safety and efficacy results of the advanced renal cell carcinoma sorafenib expanded access program in North America. Cancer.

[65] Massard C, Zonierek J, Gross-Goupil M, Fizazi K, Szczylik C, Escudier B (2010). Incidence of brain metastases in renal cell carcinoma treated with sorafenib. Ann Oncol.

